# Nurses’ Attitudes and Experiences With Generative AI in Clinical Practice: Qualitative Systematic Review

**DOI:** 10.2196/99489

**Published:** 2026-11-06

**Authors:** Mengmeng Wang, Chunyan Zhang, Naikuan Ye, Huan Li, Yue Bao

**Affiliations:** 1 Department of Peripheral Vascular Disease, The Affiliated Hospital of Shandong University of Traditional Chinese Medicine, No. 16369, Lixia District, Jinan, Shandong, China, +86 13553176792; 2 Department of Cardiology, The Affiliated Hospital of Shandong University of Traditional Chinese Medicine, Jinan, Shandong, China; 3 Department of Hepatobiliary Surgery, The Affiliated Hospital of Shandong University of Traditional Chinese Medicine, Jinan, Shandong, China; 4 Department of Nursing, Affiliated Hospital of Shandong University of Traditional Chinese Medicine, Jinan, Shandong, China

**Keywords:** artificial intelligence, generative, nurse, experiences, systematic review

## Abstract

**Background:**

The rapid advancement of generative AI (GenAI) holds transformative potential for clinical nursing practice, yet its integration also brings significant risks and uncertainties. Despite growing discourse on AI in health care, frontline nurses’ firsthand experiences, perceptions, and practical needs regarding GenAI remain largely under-synthesized in the existing literature.

**Objective:**

This systematic review aimed to synthesize qualitative evidence to clarify the core themes related to clinical nurses’ perceptions, experiences, obstacles, and requirements when using GenAI in real-world clinical settings.

**Methods:**

A systematic search was conducted across 10 electronic databases (PubMed, Embase, CINAHL, Web of Science, Cochrane Library, ProQuest Dissertations and Theses, China National Knowledge Infrastructure, VIP Information, SinoMed, and Wanfang) for records published between January 2018 and June 2026. Eligible studies included qualitative and mixed methods studies involving registered nurses that explored their perceptions and experiences of GenAI in clinical practice. Two independent reviewers (MMW and CYZ) performed study selection, quality appraisal, and data extraction, with disagreements resolved by consensus. Quality was assessed using the Joanna Briggs Institute (JBI) Critical Appraisal Checklist for Qualitative Research. Extracted data encompassed study characteristics, participant demographics, and GenAI-related contextual factors. Data synthesis followed Thomas and Harden’s 3-phase thematic synthesis: initial inductive coding generated descriptive themes, which were subsequently integrated with the Technology Acceptance Model for Artificial Intelligence in Nursing (TAM-AIN) framework to develop higher-order analytical themes. Certainty in the evidence was evaluated using the GRADE-CERQual (Grading of Recommendations Assessment, Development and Evaluation-Confidence in the Evidence from Reviews of Qualitative Research).

**Results:**

Of the 4350 initially identified records, 13 studies from 6 countries (China: n=6; Saudi Arabia: n=3; United States, Singapore, Australia, and South Korea: n=1 each) met the inclusion criteria, encompassing qualitative data from 234 registered nurses. Three main themes emerged: patterns of nurses’ engagement with GenAI (ranging from passive observation to proactive integration), multidimensional perceived threats to adoption (functional, professional, and accountability-related), and the moderating role of organizational readiness, which influenced whether these threats hindered or facilitated GenAI uptake in practice. The GRADE-CERQual assessment showed moderate to high confidence in all findings.

**Conclusions:**

Nurses’ engagement with GenAI ranges from passive observation to proactive integration, accompanied by legitimate concerns about functional reliability, professional role displacement, and accountability ambiguity. The moderating role of organizational readiness further highlights that training, tool optimization, and institutional governance remain critical yet often unmet. These findings necessitate coordinated efforts across workforce development, technological design, and regulatory frameworks. Future research should prioritize the development of clinically validated, context-aware GenAI systems that incorporate human-in-the-loop oversight, ensuring that efficiency gains do not compromise the delivery of compassionate, person-centered care.

## Introduction

The nursing profession, a cornerstone of global health care systems, faces mounting pressures, most notably chronic workforce shortages and escalating care demands. According to the World Health Organization’s “The State of the World’s Nursing 2025” report, the global shortfall of nurses and midwives was estimated at 5.8 million in 2023 and is projected to reach 4.1 million by 2030 [1]. This shortfall severely constrains the accessibility, quality, and safety of health care delivery. Moreover, population aging and the rising burden of chronic conditions continue to compound the demand for nursing services. Identifying strategies to optimize nursing productivity while safeguarding care quality has therefore become a pressing imperative.

The introduction of ChatGPT (OpenAI) in November 2022 sparked extensive discussions regarding the application of generative AI (GenAI) across various sectors. GenAI uses new types of machine-learning models to answer questions, interpret images, and deliver results in the form of newly generated original text, images, and sound—with remarkable quality and speed [2,3]. Unlike earlier AI tools that primarily identify or categorize based on established patterns, GenAI can generate entirely new and original content. These distinctive content-generation capabilities suggest its potential to transform nursing practice, particularly in areas such as drafting nursing documentation, patient education, and care plan development [4]. In clinical settings, nurses have begun using GenAI to draft handover documentation and nursing diagnoses within the electronic health records (EHRs), with both a multicenter trial across 3 hospitals and a separate 40-nurse empirical study demonstrating consistent efficiency gains, including average time reductions of approximately 40% [5,6]. GenAI has been applied to diverse clinical functions, including customized treatment planning, synthetic data generation, medical image analysis, workflow optimization, risk prediction, pandemic preparedness, and population health management. Concurrently, it may alleviate nurse burnout and reclaim time for direct patient care through automating documentation and other routine administrative tasks [7]. In medical education, GenAI shows promise for enhancing clinical training through advanced simulated patient systems that offer high-fidelity, low-cost clinical scenarios [8]. GenAI has demonstrated considerable utility; for instance, ChatGPT performs comparably to human researchers in writing introductions, with no significant differences in publication acceptability or content quality [9]. These early applications illustrate GenAI’s potential to streamline workflow and improve patient outcomes [10].

While GenAI is revolutionizing access to health information through conversational and highly personalized interactions, its capacity to generate hyper-personalized responses carries inherent risks. These include reinforcing confirmation bias, entrenching preexisting beliefs, obscuring established medical consensus, and facilitating the spread of misinformation—concerns that pose substantial public health challenges [11]. In light of these concerns, the World Health Organization has called for further research to identify risks, inform implementation strategies, and optimize the benefits of GenAI in health care settings.

Research on GenAI in nursing has evolved from broad discussions to focused inquiries in specific clinical settings and among particular user groups. Current attention centers on the sociotechnical complexities surrounding GenAI adoption—including users’ intentions, experiences, and perceptions, as well as emerging concerns and the implications of technological change [12-15]. Notwithstanding this progress, existing reviews and evidence syntheses have largely focused on educational or academic research settings [15,16]. Conspicuously absent, however, is a comprehensive synthesis that consolidates frontline nurses’ attitudes, experiences, and the practical implications of GenAI use in clinical practice. This review therefore aims to synthesize qualitative evidence on clinical nurses’ perceptions, experiences, barriers, and support needs in using GenAI. The findings will provide an evidence base for developing responsible, context-sensitive guidelines for GenAI deployment in nursing practice.

## Methods

### Protocol and Registration

This systematic review was conducted in accordance with the PRISMA (Preferred Reporting Items for Systematic Reviews and Meta-Analyses) guidelines [17] and the Enhancing Transparency in Reporting the Synthesis of Qualitative Research (ENTREQ) statement (Checklist 1 and Checklist 2) [18]. The review protocol was registered in advance with PROSPERO (International Prospective Register of Systematic Reviews; ID: CRD420251250664).

### Eligibility Criteria

Selection criteria were developed using the Population, Phenomenon of Interest, Context, and Study Design (PICoS) framework (Table 1). We included registered nurses as participants to ensure a focused understanding of their perspectives on GenAI within this professional group. In this study, GenAI was operationally defined as deep learning-based generative systems capable of producing novel, semantically meaningful content—including text, images, and audio—in response to user prompts. Eligible system architectures included, but were not limited to, large language models (LLMs), generative adversarial networks (GANs), variational autoencoders (VAEs), and diffusion models, consistent with the taxonomic framework proposed by Bandi et al [19]. This definition explicitly excluded analytical AI systems that perform pattern recognition or prediction on labeled data, as opposed to generating content. Accordingly, all rule-based and retrieval-based conversational agents were also excluded. Mixed methods studies were included only if their qualitative findings were reported separately and extractable.

**Table 1.** Inclusion and exclusion criteria.

PICoS element	Inclusion criteria	Exclusion criteria
P (Population)	RNs^a^ currently licensed and engaged in direct patient care. Studies with mixed samples are included only if data specific to RNs can be clearly isolated. Operational definition of “Registered Nurse”: for the purpose of this review, an RN is defined as an individual who has completed a recognized program of nursing education and holds a current license or registration granted by the appropriate regulatory authority in their respective country or jurisdiction, thereby authorized to practice within the legally defined scope of professional nursing, in accordance with the definitions set forth by the International Council of Nurses and the World Health Organization.	Nursing students (prelicensure). Nurse educators or researchers without concurrent clinical responsibilities. Other health care professionals (RN data not separately identifiable in mixed studies)
I (Phenomena of Interest)	Nurses’ perceptions, experiences, attitudes, beliefs, barriers, or facilitators regarding the use of GenAI^b^ in professional practice. “Use” encompasses direct or indirect engagement with GenAI tools (eg, retrieving clinical information, generating patient education materials, drafting care plans, interpreting diagnostic data, and responding to patient inquiries informed by GenAI).	Studies on general digital health or predictive AI without specific reference to GenAI. Studies focused solely on patient or caregiver perspectives.
Co (Context)	Any setting where RNs provide direct patient care (eg, hospital wards, intensive care units (ICUs), emergency departments, outpatient clinics, community health centers, and home health care). Bedside teaching and unit-based research and quality improvement are included when occurring within these settings.	Settings primarily dedicated to academic education or formal research (eg, university classrooms, simulation labs without real patients, and research institutes) where participants hold no concurrent clinical responsibilities. Social media or personal use contexts.
S (Study Design)	Primary qualitative studies (eg, phenomenology, grounded theory, ethnography, qualitative description, and case study). Mixed methods studies are included only if the qualitative component is reported separately, and findings can be independently extracted.	Quantitative studies, reviews, editorials, commentaries, protocols, and conference abstracts. Mixed methods studies where qualitative findings cannot be isolated. Non-English or Chinese full text, unretrievable full text, duplicate publications, or incomplete data.

a RN: registered nurse.

b GenAI: generative AI.

### Search Strategy

A systematic search was conducted across the following electronic databases: PubMed, Web of Science, Cochrane Library, CINAHL, Embase (including MEDLINE), ProQuest, SinoMed, China National Knowledge Infrastructure, Wanfang, VIP Chinese Science and Technology Periodical Database. The search covered the period from January 1, 2018, to June 30, 2026. The start date was set at 2018, as the transformer architecture emerged in 2017 and the first generative pretrained transformer (GPT) model was released in June 2018—events marking the onset of clinically relevant generative AI applications [2,20,21]; literature before this period was unlikely to contain meaningful content on contemporary GenAI in nursing. Our preregistered protocol originally planned a search from database inception to October 1, 2025; however, we deviated from this protocol by restricting the start date to 2018 (to ensure content relevance to the emergence of modern GenAI) and, during peer review, extending the end date to June 30, 2026, to capture the most current evidence given the rapid pace of development in this field.

The search strategy was built around 4 core conceptual domains: GenAI, nurses, and qualitative research. For each domain, we combined controlled vocabulary (eg, MeSH and Emtree) and free-text keywords, linked by Boolean operators (AND and OR), and adapted to the syntax of each database. In addition, we manually screened the reference lists of all included studies to complement the electronic database searches. Detailed search strategies for each database are available in Multimedia Appendix 1.

### Study Selection and Screening

All retrieved records were imported into NoteExpress (Beijing Aegean Software Co., Ltd.) for deduplication and screening. Study selection was conducted independently by two reviewers (MMW and CYZ). Initially, titles and abstracts were screened against eligibility criteria. Potentially eligible records then proceeded to full-text review. Disagreements at each stage were resolved by discussion or, if necessary, by consulting a third reviewer (YB).

### Methodological Quality Assessment

Two reviewers (MMW and CYZ) independently assessed the methodological quality of the included studies using the Joanna Briggs Institute (JBI) Critical Appraisal Checklist for Qualitative Research [22]. The checklist consists of 10 items assessing aspects such as congruity between the research methodology and various study components. The appraisal results for each item included “Yes”, “No”, “Unclear”, and “Not Applicable”. A response of “Yes” was scored as 1 point, whereas “No” and “Unclear” were scored as 0 points. “Not Applicable” was excluded from the denominator when calculating the percentage score. The criteria for determining the risk of bias were as follows: a score of at least 7 out of 10 was classified as low risk, 5 to 6 out of 10 as moderate risk, and less than 5 out of 10 as high risk[23]. Discrepancies between reviewers were resolved through discussion with a third reviewer (YB). Only studies rated as low or moderate risk were included. All 13 studies [24-36] met this inclusion threshold (Multimedia Appendix 2).

### Data Extraction, Synthesis, and Theoretical Framing

Data were extracted systematically by 2 reviewers (MMW and CYZ) using a standardized form. The extracted information included the authors, the year of publication of the literature, country or region, research methodology, subjects of the study, phenomenon of interest, situational factors, type of GenAI and key features, and key findings related to attitudes and experiences (Multimedia Appendix 3). Consensus on extraction was reached through discussion, with any disagreements arbitrated by a third reviewer (YB).

For the synthesis, we used the 3-stage thematic synthesis approach proposed by Thomas and Harden [37]. Phases 1 and 2 involved inductive line-by-line coding to generate descriptive themes; Phase 3 used a post hoc interpretive lens—the Technology Acceptance Model for Artificial Intelligence in Nursing (TAM-AIN) proposed by Ramadan et al [38]—to elevate these descriptive themes into higher-order analytical themes. The rationale for this analytical choice is as follows. The TAM-AIN extends Davis’ classic TAM [39,40], which identifies perceived usefulness and perceived ease of use as primary determinants of technology adoption, by incorporating three additional constructs specifically relevant to nursing: ethical alignment (eg, concerns about patient privacy and the preservation of human dignity), organizational readiness (eg, institutional support, infrastructure, and training), and perceived threats to professional autonomy (eg, fears of job displacement or deskilling). We selected this framework because it is domain-specific and empirically grounded in nurses’ perspectives, offering a coherent structure to organize our descriptive themes into higher-order analytical insights. Crucially, this model was not used to design data collection instruments or to guide initial coding; it was applied retrospectively—after descriptive themes had been generated—solely to enrich interpretation and to generate novel conceptual insights, rather than to constrain or predetermine our analysis.

Two researchers (MMW and CYZ) independently performed initial line-by-line coding using NVivo 15 (Lumivero). Our preregistered protocol specified NVivo 11; however, during the review process, we updated to NVivo 15 to take advantage of improved stability and interface enhancements. This technical upgrade did not affect the coding structures or analytical outputs. Disagreements in coding or theme development were resolved through discussion, with consensus reached through team deliberation. To enhance coding reliability, the research team held regular coding review meetings every 2 weeks to systematically review all codes and reach consensus.

The analysts were clinical nurses who used GenAI in their daily work and held positive attitudes toward the technology. We acknowledge that this may have predisposed us to emphasize positive experiences and benefits while potentially under-examining concerns and negative experiences. To minimize bias, the team engaged in reflexive dialogues throughout the analysis. All codes and themes were independently cross-checked, and disagreements were debated until consensus was reached.

### Assessing Confidence in the Findings

We used the GRADE-CERQual (Grading of Recommendations Assessment, Development and Evaluation-Confidence in the Evidence from Reviews of Qualitative Research) approach to assess the level of confidence in the findings of this qualitative meta-synthesis [41]. GRADE-CERQual provides a systematic and transparent framework for assessing confidence in individual review findings based on 4 components: methodological limitations, coherence, adequacy of data, and relevance. For each review finding, 2 reviewers (MMW and CYZ) independently assessed the four components, with disagreements resolved through discussion or arbitration by a third reviewer (YB), and the overall confidence was rated as high, moderate, low, or very low using the GRADE-CERQual approach. A rating of high confidence indicates that it is highly likely that the review finding is a reasonable representation of the phenomenon of interest.

## Results

### Overview

A total of 4350 studies were initially identified, comprising 4346 from database searches and 4 through reference list screening. After duplicates were removed, 2935 unique records underwent title and abstract screening, yielding 67 articles for full-text review. After full-text assessment, 54 records were excluded, leaving 13 studies [24-36] that met all inclusion criteria (Figure 1).

**Figure 1.**
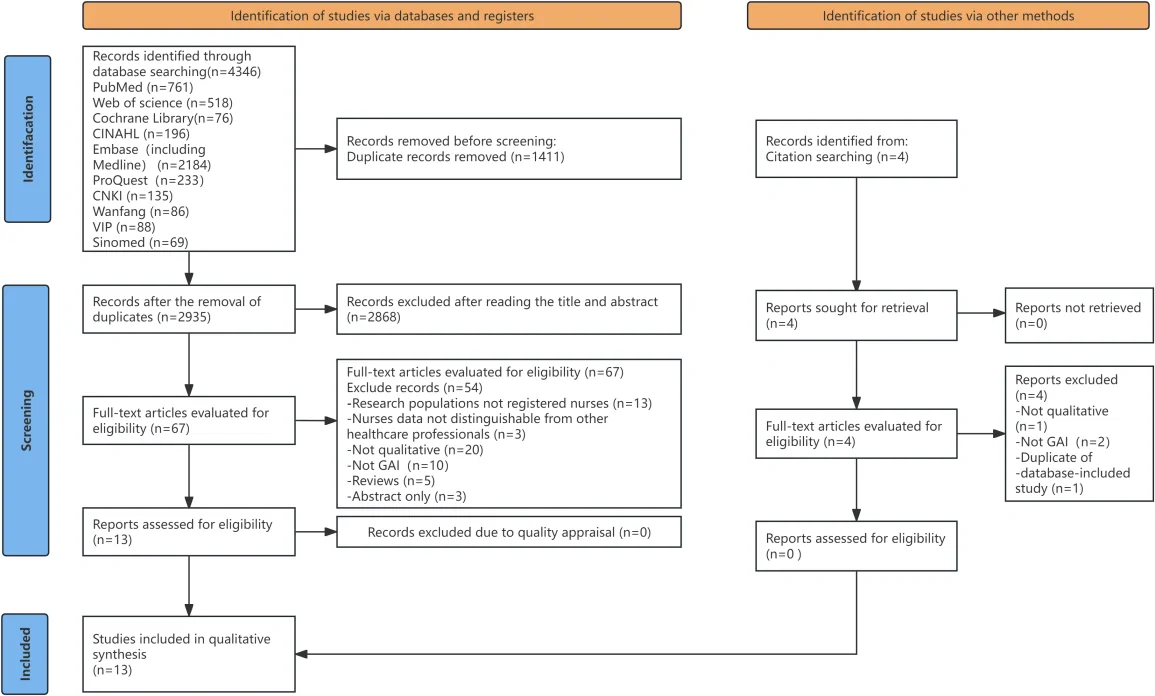
Literature screening process. GAI: generative AI.

All of the included studies (n=13) [24-36] were rated as having a low risk of bias, with detailed quality assessment results presented in Multimedia Appendix 2. While study design and ethical considerations were generally robust, a common limitation was a lack of explicit reporting on researcher reflexivity and positioning. The 13 included studies [24-36] were published between 2023 and 2026 and were conducted across 6 countries: China (n=6) [26,28,30,33-35], Saudi Arabia (n=3) [27,29,36], and one each from the United States [32], South Korea [31], Australia [24], and Singapore [25]. The studies took place in various health care and academic settings, including general hospitals, teaching hospitals, specialized cancer centers, and university-affiliated simulation centers. In total, these studies comprised qualitative data from 234 registered nurses working in various specialties, including intensive care, oncology, pediatrics, neonatology, and general wards. One study [24] used a mixed methods design, while the other 12 studies [25-36] were qualitative.

### Confidence in the Review Findings

Confidence in the review findings ranged from high to moderate. Reasons for downgrading were due to concerns regarding coherence and/or adequacy of data. Among the 9 subthemes, 4 were rated as moderate confidence due to minor to moderate concerns regarding coherence and/or adequacy of data (Table 2).

**Table 2.** Summarized review categories and GRADE-CERQual (Grading of Recommendations Assessment, Development and Evaluation-Confidence in the Evidence from Reviews of Qualitative Research) assessment.

Findings	References	GRADE-CERQual assessment of confidence	Explanation of overall assessment
Subtheme 1.1: Passive Observation—Novice Users’ Cautious and Distant Stance	4 studies (Liu et al [28]; Tong et al [33]; Wang et al [34]; Yin et al [35])	Moderate confidence^a^	No or very minor concerns regarding methodological limitations, minor concerns regarding coherence, moderate concerns regarding adequacy, and no or very minor concerns regarding relevance.
Subtheme 1.2: Pragmatic Usage—Intermediate Users’ Task-Oriented Adoption	13 studies (Nazir et al [24]; Shankar et al [25]; Qu et al [26]; Alanzi et al [27]; Liu et al [28]; Artuwaili et al [29]; Kang et al [30]; Han et al [31]; Bautista et al [32]; Tong et al [33]; Wang et al [34]; Yin et al [35]; Temsah et al [36])	High confidence^b^	No or very minor concerns in all four domains.
Subtheme 1.3: Proactive Integration—Advanced Users’ Collaborative and Transformative Engagement	10 studies (Nazir et al [24]; Shankar et al [25]; Qu et al [26]; Liu et al [28]; Artuwaili et al [29]; Kang et al [30]; Han et al [31]; Bautista et al [32]; Tong et al [33]; Wang et al [34])	High confidence^b^	No or very minor concerns in all four domains.
Subtheme 2.1: Functional Deficits—Concerns Over Inaccuracy, Insecurity, and Instability	12 studies (Shankar et al [25]; Qu et al [26]; Alanzi et al [27]; Liu et al [28]; Artuwaili et al [29]; Kang et al [30]; Han et al [31]; Bautista et al [32]; Tong et al [33]; Wang et al [34]; Yin et al [35]; Temsah et al [36])	High confidence^b^	No or very minor concerns in all four domains.
Subtheme 2.2: Professional Erosion—Threats to Critical Thinking, Compassion, and Identity	11 studies (Nazir et al [24]; Shankar et al [25]; Qu et al [26]; Alanzi et al [27]; Liu et al [28]; Artuwaili et al [29]; Han et al [31]; Bautista et al [32]; Tong et al [33]; Wang et al [34]; Yin et al [35])	High confidence^b^	No or very minor concerns in all four domains.
Subtheme 2.3: Accountability Ambiguity—Blurred Boundaries of Legal and Ethical Responsibility	6 studies (Qu et al [26]; Alanzi et al [27]; Han et al [31]; Bautista et al [32]; Tong et al [33]; Yin et al [35])	Moderate confidence^a^	No or very minor concerns regarding methodological limitations, no or very minor concerns regarding coherence, moderate concerns regarding adequacy, no or very minor concerns regarding relevance.
Subtheme 3.1: Cultural Preparedness—Fostering Training and Social Endorsement	8 studies (Nazir et al [24]; Shankar et al [25]; Liu et al [28]; Artuwaili et al [29]; Kang et al [30]; Han et al [31]; Bautista et al [32]; Wang et al [34])	Moderate confidence^a^	No or very minor concerns regarding methodological limitations, no or very minor concerns regarding coherence, minor concerns regarding adequacy, no or very minor concerns regarding relevance.
Subtheme 3.2: Technical Preparedness—Enhancing Usability, Applicability, and Response Precision	10 studies (Nazir et al [24]; Shankar et al [25]; Qu et al [26] Alanzi et al [27]; Liu et al [28]; Artuwaili et al [29]; Kang et al [30]; Han et al [31]; Bautista et al [32]; Tong et al [33])	High confidence^b^	No or very minor concerns in all four domains.
Subtheme 3.3: Institutional Preparedness—Strengthening Oversight, Regulation, and Funding	6 studies (Nazir et al [24]; Shankar et al [25]; Qu et al [26]; Artuwaili et al [29]; Han et al [31]; Bautista et al [32])	Moderate confidence^a^	No or very minor concerns regarding methodological limitations, minor concerns regarding coherence, moderate concerns regarding adequacy, no or very minor concerns regarding relevance.

a It is likely that the review finding is a reasonable representation of the phenomenon of interest.

b It is highly likely that the review finding is a reasonable representation of the phenomenon of interest.

Three overarching themes were identified: engagement patterns across adoption stages, multidimensional perceived threats, and the moderating role of organizational readiness; these comprised 9 subthemes grouped respectively as novice, intermediate, and advanced user patterns; functional, professional, and accountability concerns; and cultural, technical, and institutional preparedness. The findings suggest that perceived threats undermine perceived usefulness and ease of use while amplifying professional autonomy concerns, functioning as primary barriers. Organizational readiness moderates this dynamic: high preparedness alleviates threats and promotes proactive engagement, whereas low readiness reinforces obstacles and sustains conditional use.

### Theme 1: Patterns of Nurses' Engagement With GenAI

#### Overview

Nurses’ attitudes and practices regarding GenAI were described as forming a continuum ranging from passive observation to active integration, reflecting varying levels of engagement with the technology. These patterns were perceived by participants to be influenced by varying levels of perceived usefulness, perceived ease of use, perceived threats to professional autonomy, ethical congruence, and organizational readiness. The synthesis identified three distinct engagement patterns among nurses within clinical settings: novice users, who were characterized by a passive, wait-and-see stance; intermediate users, who acted as pragmatic adopters; and advanced users, who described actively seeking deeper integration of GenAI into clinical workflows—for example, in nursing education and patient care.

#### Subtheme 1.1: Passive Observation—Novice Users' Cautious and Distant Stance

Four included studies [28,33-35] identified novice users who acknowledged GenAI’s rapid advancement but described themselves as remaining at the periphery, adopting a cautious “wait-and-see” stance. Findings highlighted several interrelated barriers associated with this cautious stance: lack of training, limited hands-on experience with GenAI, and concerns about its clinical reliability. These concerns were frequently centered on fears that AI may erode the humanistic essence of nursing and compromise patient care, as well as anxieties about data privacy and security. Theoretically, this group reported very low perceived ease of use, which was linked in their accounts to low confidence in using GenAI, and heightened perceived threats to professional autonomy. Across these accounts, participants frequently described these interconnected perceptions alongside distrust, perceived limited usefulness, and a predominantly passive, detached posture toward GenAI.

For example, one nurse stated hesitantly:

Since I’m not engaged in research, why should I bother learning about these matters?

(N12, regarding the application of ChatGPT-like language models in medicine [35])

Another nurse shared:

I’m not yet proficient in using AI tools, and I often feel that my AI-generated scenarios are not as well-structured as those created by others. I’m concerned about potential challenges in scaling AI adoption.

(N11, ICU novice simulation instructors who participated in a simulation instructor training program incorporating Gen AI for case design [28])

#### Subtheme 1.2: Pragmatic Usage—Intermediate Users' Task-Oriented Adoption

Across all 13 included studies [24-36], intermediate users used GenAI as a tool to enhance efficiency and support their professional practice. Their use of GenAI was predominantly task-oriented, focusing on alleviating administrative burden. Key applications included automating clinical documentation, generating handover summaries, drafting personalized patient education materials, and staying updated with recent evidence for practice or research. They regarded GenAI not as a replacement but as a valuable complementary tool, perceived as streamlining workflows, minimizing manual data entry, and freeing time for direct patient care. Beyond operational gains, they viewed GenAI as a resource for professional development, linking it to skill acquisition, reduced documentation errors, and what they perceived as improved patient outcomes—a pattern that was consistent with their high ratings of perceived usefulness. However, their adoption remained conditional. They emphasized the need for ongoing training and robust human oversight, while also expressing concerns about sociodemographic biases in AI-generated content and ethical issues.

For example, one participant stated:

I do see generative AI being one of the underlying software in avatars that patients will speak to in a digital setting, whether through video chats, phone calls, or texts, in which we offload some of the more time-intensive and repetitive conversations that nurses have.

(Practitioner 8, informatics nurse administrator, on the use and implications of GenAI tools in nursing education and practice [32])

A second participant stated:

When I need to design a bulletin board for infant health education, it can perform hotspot analysis and generate a preliminary outline for me. I can then augment it with my professional knowledge, thereby enhancing work efficiency.

(N5, a pediatric nurse, on her experience with GenAI [34])

Another participant remarked:

When the AI flags potential respiratory distress, I don’t just change settings blindly. I check the baby’s color, muscle tone, and response. The AI points me in a direction, but I still make the call.

(P22, a neonatal nurses’ experiences with GenAI in clinical decision-making [29]

#### Subtheme 1.3: Proactive Integration—Advanced Users' Collaborative and Transformative Engagement

In 10 included studies [24-26,28-34], participants were characterized as pioneers in integrating GenAI into nursing practice. They moved beyond basic tool use to engage GenAI as a collaborative partner in clinical practice, demonstrating high ethical congruence and proactively embracing technological change. Mastery of prompt engineering was a core competency that seemingly enabled them to optimize AI outputs for clinical relevance. Advanced users viewed the inevitable evolution toward AI adoption and actively redefined their professional roles and skillsets to work effectively with AI. This proactive identity shift apparently transformed potential threats to professional autonomy into developmental opportunities. A guiding principle was that humanistic care should govern technology use, ensuring that AI augments clinical judgment rather than replaces it. This engagement was described as fostering a reciprocal development cycle: interacting with GenAI expanded cognitive perspectives, was seen as a catalyst for continuous learning, and was linked to deeper expertise for guiding AI effectively. Driven by strong perceived usefulness across multiple domains, they explored advanced applications such as data analysis and documentation to enhance diagnostic reasoning and free time for direct patient care. Ultimately, this appeared to reflect a dual internalization—at both cognitive and behavioral levels.

For example, one nurse stated:

In many cases, I feel that my thinking is not comprehensive enough, especially as I have worked in a clinical environment for many years and can have some inherent thoughts that limit my thinking. By interacting with GAI, I feel that my thinking and perspective have been broadened.

(FG3-N23, clinical nurse, experience with GenAI training for research support [33])

Another nurse stated:

In the past, we spent a great deal of time searching for literature and organizing data. Now, we must learn how to guide AI to generate valuable content and to propose directions for further research. We have become more like helmsmen of the research direction, while AI serves as the engine that provides the power.

(N14, clinical nurse, experience with the perceptions and application of GenAI [26])

A third nurse added:

Sometimes, the AI confirms what I’m already suspecting, and other times, it challenges me to reconsider. Either way, it ensures I’m never complacent.

(P29, Neonatal nurses’ experiences with GenAI in clinical decision-making [29])

### Theme 2: Nurses' Multidimensional Perceived Threats to GenAI Adoption

#### Overview

Nurses’ perceived threats regarding GenAI in terms of 3 core dimensions: functional (accuracy and confidentiality concerns), professional (threats to humanistic care and professional autonomy), and accountability-related (uncertainties about legal and professional responsibility). In addition, they reported a cautiously optimistic orientation, characterized by guarded enthusiasm tempered by concerns, which they discussed alongside these perceived threats.

#### Subtheme 2.1: Functional Deficits—Concerns Over Inaccuracy, Insecurity, and Instability

Functional deficits emerged as a key concern across 12 studies [25-36], wherein nurses expressed significant reservations regarding the reliability and infrastructural support of GenAI. These concerns, which align with the perceived threats to functionality posited by the TAM-AIN framework, were primarily categorized into three domains: (1) output accuracy and clinical validity—specifically, the risk of “hallucinations,” limited ability to handle complex clinical contexts, and the generation of misleading information that could harm patients or delay care; (2) privacy and data security—fears that GenAI could increase the risk of patient data breaches and information leakage, along with concerns about the need for strict safeguards to prevent misuse; (3) system stability and infrastructural support—worries about technical failures, connectivity issues, and inadequate IT support disrupting workflows. Overall, nurses perceived these concerns as significant barriers that could impede their acceptance and sustained use of GenAI.

One nurse remarked:

I find it difficult to use GenAI efficiently without a research foundation. For example, when GenAI provides citations or recommends statistical methods, I need a research foundation to judge the accuracy of the content. Without a research base, GenAI may mislead me into misusing the information.

(FG1-N4, nurse’s perceptions and experiences of GenAI training to support research [33])

As one nurse noted:

I feel confident when I know there’s IT support at hand. Without it, the AI can become a source of frustration rather than insight.

(P26, neonatal nurses’ experiences with GenAI in clinical decision-making [29])

Another nurse stated:

I’m hesitant to try it because I’m not sure where the information goes. Even if we don’t use names, I worry someone could still trace it back to a patient or misuse this data.

(P15, Exploring Early Perceptions and Experiences of ChatGPT in Pediatric Critical Care [36])

#### Subtheme 2.2: Professional Erosion—Threats to Critical Thinking, Compassion, and Identity

As described in 11 papers [24-29,31-35], nurses also worried about the cognitive and professional implications of GenAI adoption, reflecting TAM-AIN role threats. Concerns clustered into 3 interrelated areas:

Erosion of autonomous and critical thinking—they perceived that GenAI might inhibit users’ critical thinking, with particular concerns that overreliance could foster dependency and impair independent clinical judgment, especially among younger nurses. Nurses emphasized that AI should serve as an augmentative tool to support traditional nursing practice and a learning aid to complement—not substitute—clinical reasoning

Threats to humanistic care, professional identity, and the nurse-patient relationship—although some acknowledged that reduced documentation burden could free up time for bedside care, others worried that technological dependency would reduce direct patient contact. They feared that misuse of GenAI could erode patient trust, hinder rapport-building, and raise ethical dilemmas about whether patients are ultimately interacting with a machine or a human. Nurses expressed ambivalence, as they saw it as potentially enhancing humanistic care in administrative tasks, while challenging their caring role in patient-interactive tasks.

Nurses indicated that GenAI could undermine the humanistic foundation of nursing practice. Given its inherent difficulty in establishing genuine trust with patients and its limitations in emotional understanding, nurses were concerned that GenAI cannot authentically experience or convey empathy or compassion. This may compromise personalized care and result in a deficit of humanistic elements. Furthermore, nurses perceived that traditional nursing models are being challenged. As medical and nursing work grows increasingly complex, the professional identity of nurses in the AI era has also become more multifaceted. They expressed anxiety that core nursing roles might be diminished or replaced by AI and have begun to reflect on how to address this impact, as well as on the future direction of the profession.

One nurse stated:

No matter how well the machine displays information and reacts, it’s not the same as direct communication. If nurses are only administering medication without interaction, it would likely hinder rapport building.

(An oncology nurse’s perceptions for GenAI [31])

Another nurse observed:

I’ve seen many technologies come and go. Some helped, some created more problems. My concern is that younger nurses might start trusting the machine too much. Nursing is about thinking, observing, connecting things that don’t obviously connect. Can AI do that? I don’t think so, not yet.

(RN02, an experienced nurse expressed greater caution regarding GenAI-enabled shift handover innovation [25])

#### Subtheme 2.3: Accountability Ambiguity—Blurred Boundaries of Legal and Ethical Responsibility

A total of 6 papers [26,27,31-33,35] reported that nurses articulated significant concerns regarding legal and ethical ambiguity—concerns about accountability in the event of clinical errors, difficulties in determining responsibility when mistakes occur, transparency in patient interactions (eg, disclosing AI-generated responses), and ethical dilemmas regarding whether patients are ultimately interacting with a machine or a human. In addition, nurses raised concerns about the blurred attribution of academic contributions and perceived threats to academic integrity when using GenAI in educational or research settings. Nurses also called for stronger governance, including clearer legal frameworks, ongoing monitoring, and stronger institutional guidance to ensure responsible use. They perceived regulatory and ethical considerations as critical factors in the effective implementation of GenAI.

One oncology nurse questioned:

From an ethical standpoint, if AI produces incorrect results, can the consequences be solely placed on the patient? Who should be held accountable- the manufacturer of the device or the developer of the software?

(A nurse’s perceptions of GenAI [31])

A second nurse commented:

I saw that the journals under the Chinese Medical Association had issued a joint statement that articles using GenAI writing would not be accepted for publication. Then I thought, is there any point in learning how to use it? If I write a part of an article using GenAI but do not know how to label or describe it, will that make me a researcher defaulter?

(FG1-N14, Perceptions and experiences of GenAI training to support research [38])

A third nurse with research experience observed:

When using AI to assist in writing papers, even with substantial modification and deepening of the content, it remains difficult to define the boundary of personal contribution. This uncertainty diminishes the sense of achievement in research work.

(N6, a clinical nurse regarding perspectives on GenAI [26])

### Theme 3: The Moderating Role of Organizational Readiness on GenAI Adoption

#### Overview

Nurses described their perceptions of organizational readiness in terms of three interconnected domains. In terms of cultural readiness, nurses consistently voiced a compelling need for enhanced training and education to build competency and foster greater societal acceptance of GenAI-involved nursing care. Regarding technical readiness, they discussed a need for optimized, clinically-tailored tool development to ensure relevance and utility, with more specialized and precise responses expected from GenAI systems. With respect to institutional readiness, they identified a need for proactive organizational strategy and support to enable safe and effective implementation, encompassing improved technical oversight, personnel preparation, and regulatory frameworks. Participants described organizational readiness as influencing their acceptance and sustained use of GenAI. They reported that higher levels of preparedness across cultural, technical, and institutional domains were associated with greater openness to adoption, while deficiencies in any dimension were noted as potential challenges. Across these accounts, participants identified addressing these interconnected needs as fundamental to successful GenAI integration into nursing practice.

#### Subtheme 3.1: Cultural Preparedness—Fostering Training and Social Endorsement

In eight studies [24,25,28-32,34], nurses described cultural readiness in terms of two interrelated dimensions: internal workforce capacity-building and external societal acceptance. Internally, nurses expressed a strong unmet need for GenAI training. Many reported limited understanding of GenAI functionality, which they identified as a barrier to trust and willingness to use these tools. They called for dedicated, practical training and integration of GenAI-related knowledge into nursing curricula and continuing professional education, which they viewed as necessary for informed decision-making and safe, effective use. Externally, cultural readiness was seen as fostering broader societal acceptance. Nurses recognized that local cultural beliefs and values influence AI adoption in clinical practice and suggested that hospital- and community-level advocacy would be needed to enhance patient and public acceptance of GenAI involvement in nursing care.

One nurse observed:

Saudi Arabia’s national push for digital health support makes us more willing to embrace AI. It feels like we are part of a larger modernization effort.

(P 30, a neonatal nurse’s experience with GenAI in clinical decision-making [29])

A second nurse remarked:

I don’t think that in five years from now, the way we practice today will be the way we practice tomorrow. New nurses coming in need to have a baseline understanding of how to prompt these [GenAI] systems to get an output that would be helpful in a clinical environment.

(Practitioner 11, nursing professional development nurse administrator view on the use and implications of GenAI tools on nursing education and practice [32])

#### Subtheme 3.2: Technical Preparedness—Enhancing Usability, Applicability, and Response Precision

The technical preparedness was reported in 10 papers [24-33], technical readiness encompasses not only system usability and workflow integration but also the expansion of potential applications, the development of more specialized content, and the delivery of more precise responses. Participants expressed that current GenAI tools require significant optimization to meet the nuanced demands of clinical practice. They emphasized the need for improved interoperability with existing systems such as the electronic health record (EHR) and more user-friendly interfaces to enhance usability and seamless workflow integration. They described potential applications for GenAI, including substituting high-risk activities (eg, chemotherapy preparation), assisting with vital signs observation, and guiding patient specimen collection, as well as simulating patient interactions, conducting emergency drills, and training new nurses. Furthermore, they advocated for the development of nursing-specific GenAI systems, specialized databases or corpora, and institutionally tailored solutions to enhance clinical relevance and professional rigor. In addition, nurses desired more accurate and context-aware responses from GenAI, with the ability to capture complete information, perform structured data processing, and aggregate and summarize complex datasets, which they viewed as providing more fine-grained and precise answers to support clinical decision-making. Nurses described addressing these technical readiness needs as important for their acceptance and sustained use of GenAI.

One informatics nurse observed:

Generative AI tools are not going to be able to be maximally helpful unless they are linked to the patient’s electronic health record, so that inferences can be made about very specific patient factors, because the more specific the recommendations are, the more actionable and helpful they are for nurses.

(Practitioner 8, informatics nurse administrator, on the use and implications of GenAI tools in nursing education and practice [32])

A second nurse remarked:

Could we develop a decision support system using GenAI to input patient temperature data and other vital signs . This system would help us train new nurses and reduce our workload.

(FG1-N4, perceptions and experiences of GenAI training to support research [33])

A third nurse asked:

Is there a corpus for the nursing profession, or some database, that would allow us to search more accurately for future nursing research knowledge for nurses? That way, the information nurses receive will be more reliable.

(C3, a clinical nurse, regarding researchers’ perceptions of GenAI [30])

#### Subtheme 3.3: Institutional Preparedness—Strengthening Oversight, Regulation, and Funding

Across six reviewed papers [24-26,29,31,32], institutional readiness was characterized by technical oversight, clear legal and regulatory frameworks, and adequate staffing and funding allocation. Nurses expressed that organizational decisions and support structures shaped the adoption and effective use of GenAI software. They described the organization’s stance and resource levels as factors that influenced the depth and quality of GenAI integration. They emphasized the necessity of technical oversight during GenAI application, including routine monitoring, evaluation, feedback mechanisms, and the essential role of human supervision. Furthermore, they called for clear regulations and guidelines from hospital management regarding which AI programs are permitted for use within the system and called for the prompt establishment of standardized guidelines for the responsible use of GenAI in research contexts. They also expressed a desire to reduce cumbersome procedural approvals when using GenAI within the organization. In terms of personnel and funding, participants noted that sufficient staffing was needed for GenAI to function effectively and stressed that the implementation of GenAI requires adequate financial support. They also hoped for active organizational support in developing or procuring customized GenAI solutions aligned with specific institutional missions and clinical workflows, which they viewed as a potential strategy to safeguard the confidentiality of protected health information.

One oncology nurse manager observed:

There was information put out [by the hospital management] about which AI programs we are allowed to use in our system. Right now it’s only Microsoft [Copilot].

(Practitioner 1, oncology nurse manager, on the use and implications of GenAI tools in nursing education and practice [32])

A second nurse emphasized:

Legal support is necessary. We need to demonstrate that we are well-prepared in case legal issues arise. Only then can nurses use the technology with peace of mind. If the responsibility falls entirely on the nurses, they may not use it.

(A nurse’s perception of GenAI [31])

A third informatics nurse added:

My workplace is very vocal about wanting to be the number one global leader for digital health. For example, I got a $1,000,000 grant to work on this.

(Practitioner 8, informatics nurse administrator, on the use and implications of GenAI tools in nursing education and practice [32])

## Discussion

### Principal Findings

This synthesis of 13 qualitative studies [24-36] (234 registered nurses, 6 countries) characterizes nurses’ adoption of GenAI in clinical settings. Three overarching themes and 9 subthemes emerged: engagement patterns (novice, intermediate, and advanced users), multidimensional perceived threats (functional, professional, and accountability), and the moderating role of organizational readiness (cultural, technical, and institutional). These findings not only consolidate existing evidence but also extend the TAM-AIN framework, as we elaborate below.

### Theoretical Contributions: An Extended TAM-AIN Framework

The findings support and extend the TAM-AIN framework [38] by showing that organizational readiness emerged as a key shaping factor, not merely an antecedent. This aligns with Shankar et al’s [25] finding that nurses exhibit cautious optimism and conditional acceptance. At the same time, the findings advance their work by interpreting these “conditions” as concrete threat and readiness dimensions, making the concept more relevant for intervention design. These contributions provide theoretical guidance for future research and clinical management, and point to targeted strategies in training, technical optimization, and policy formulation.

Building on these empirical insights, we propose an integrated analytical framework (Figure 2) that extends the TAM-AIN. This framework captures the dynamic interplay through which perceived usefulness, perceived ease of use, ethical congruence, organizational readiness, and multidimensional perceived threats collectively shape nurses’ adoption behaviors and differentiates three distinct user types. The framework advances the TAM-AIN in 2 key ways. First, it differentiates the formerly unified construct “perceived threat to professional autonomy” into three distinct dimensions—erosion of humanistic care, ambiguity in legal liability, and degradation of cognitive skills—each of which emerges as a distinct barrier to technology uptake. Second, organizational readiness is reconceptualized not as a unified construct, but as three distinct pathways—technical, institutional, and cultural preparedness—that appear to shape adoption by shaping perceptions of ease of use, mitigating perceived threats, and reinforcing ethical congruence. Taken together, these conceptual refinements illustrate how individual perceptual dynamics and organizational ecologies interact to shape nurses’ use of GenAI, the barriers they encounter, and the unmet needs they express in clinical practice.

**Figure 2.**
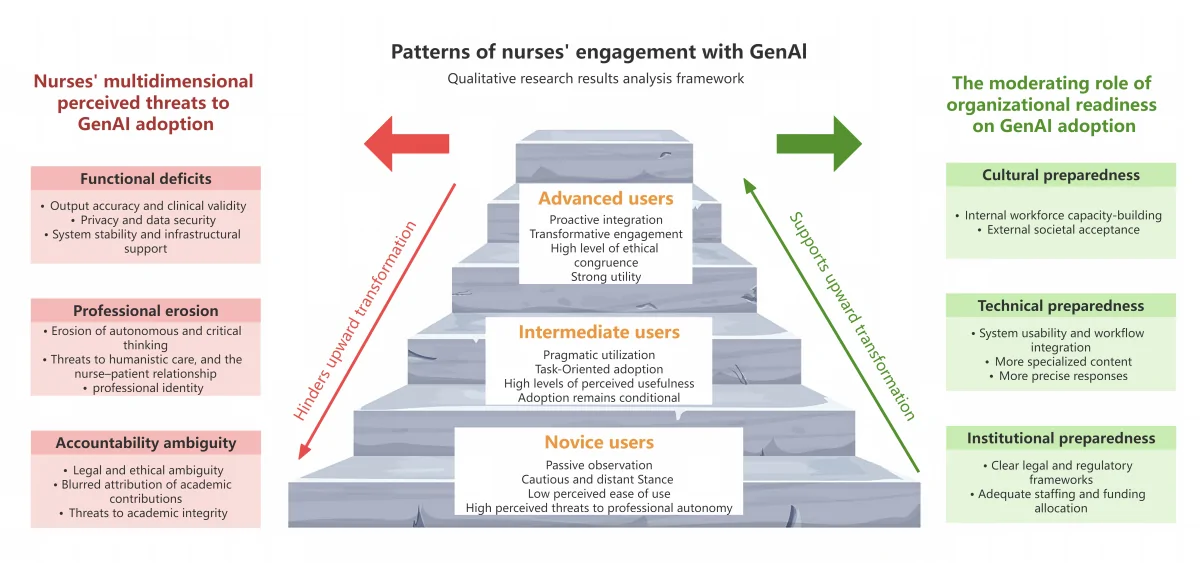
Qualitative research analysis framework. GenAI: generative AI.

### GenAI in Health Care: An Inevitable Trend Requiring Proactive Engagement

The rapid advancement of GenAI over recent years represents a technological transformation that is increasingly penetrating the high-stakes medical field [42]. Systematic reviews have highlighted its potential to enhance health care efficiency through streamlining workflows, reducing costs, and personalizing care [43]. Surveys indicate generally positive attitudes among health care professionals. For example, a study of 902 professionals (54.9%, 495/902 of whom were nurses) found that 56.1% (506/902) felt comfortable using AI in practice, and 72% (649/902) believed it would improve future health care [44]. In a similar vein, a study of Turkish nurses found that 84.4% (243/288) believed nurses should be informed about ChatGPT and AI, viewing it as beneficial for professional development and patient care [45].

These positive attitudes align with our finding that nurses exhibited varying levels of engagement with GenAI, reflecting a continuum from novice to intermediate to advanced users. As GenAI becomes more widespread, the proportion of passive novice users will likely diminish, while intermediate and advanced users drive its application, catalyzing transformative changes in nursing practice. Notably, advanced users engaged with GenAI as a collaborative partner in clinical practice, actively redefining their professional roles to adapt.

### Navigating Challenges: A Call for Critical Development and Prudent Implementation

Current evidence suggests that GenAI does not yet match expert human clinical judgment. For example, one comparative study [46] found that experienced community nurses outperformed a leading GenAI system in diagnostic accuracy and clinical decision-making in simulated scenarios, with AI responses being faster but often verbose and containing unnecessary information. Other research has noted tendencies toward indecisiveness and over-triage in AI tools [47], advising caution against premature deployment as a nurses’ digital aide. Our review confirms these technical and reliability concerns and extends them by identifying broader threats perceived by nurses. These fall into three categories: functional deficits (inaccuracy and data insecurity), professional erosion (critical thinking, compassion, and professional identity), and ambiguity in legal and ethical responsibility.

Moreover, deeper familiarity with GenAI appears to temper initial optimism with greater critical awareness. A study of nursing educators’ perceptions of AI chatbots found that frequent users had a more nuanced understanding of the advantages and limitations, whereas infrequent users expressed fewer concerns—a pattern consistent with the TAM [13]. Our findings show that nurses’ specific worries—about misinformation, erosion of humanistic care, and legal and ethical dilemmas—become more clearly articulated with deeper engagement.

Beyond the immediate concern of erosion of humanistic care, the literature increasingly points to the possibility that GenAI may fundamentally reshape nursing professional identity. This is not merely a workflow adjustment but a profound challenge to professional identity. One qualitative study reported that 87.9% (29/33) of neonatal nurses identified “Professional Identity in the AI Era” as a key theme, with 84.8% (28/33) reporting shifting role boundaries [29]; likewise, an integrative review identified "Role Boundaries, Ethics, and Trust" as a core theme [21]. Our synthesis further delineates three interrelated pathways of professional erosion: threats to critical thinking (via overreliance), to compassionate care (via relationship erosion), and to professional identity (via role diminishment). This 3-part operationalization provides a more actionable framework than prior studies that treated identity threat as a monolithic concern.

### Building Organizational Readiness: A Roadmap for Proactive Implementation and Nurse-Led Co-Design

The unmet needs identified in this review-targeted education, clinically optimized tools, and robust institutional governance-echo broader recommendations in the literature [44,48]. Importantly, our findings position organizational readiness as a moderating factor: higher levels of cultural, technical, and institutional preparedness alleviate threat perceptions and enhance confidence, thereby enabling more proactive engagement, whereas deficiencies in any dimension sustain conditional use. A systematic review [49] of AI in nursing further found that adoption is driven by perceived usefulness, self-efficacy, and enabling environments, with anxiety and demographics influencing engagement. An integrative review [21] of GenAI in clinical nursing practice reinforced these findings, finding that “safe and effective integration will require rigorous nurse training, robust governance, transparent labeling of AI-generated content, and ongoing evaluation”—elements that correspond directly to our three dimensions of organizational readiness.

Previous work has proposed strategies including developing multimodal systems integrated with real-time data (eg, EHRs), using emotional computation, and establishing comprehensive ethical and policy frameworks [10]. Specifically, we recommend: (1) prioritizing the development of clinically validated, context-specific models to ensure reliability and safety; and (2) embedding a human-in-the-loop principle in tools to preserve nursing oversight and professional autonomy. Our synthesis identifies accessible IT support as a decisive factor separating conditional users from proactive adopters. The human-in-the-loop model, in which educators or clinicians validate GenAI outputs, has been proposed as a key framework for cultivating nurses who use technology critically and ethically while maintaining competent, human-centered care [50]. Importantly, our findings extend this model by specifying that accessible IT support is a nonnegotiable prerequisite: without it, even the best-designed human-in-the-loop framework fails, because nurses lack the confidence to engage; (3) adopting a systematic implementation roadmap that synchronizes policy, training, and nurse-led co-design. These steps are crucial for aligning GenAI development with the core values and practical realities of nursing.

### Limitations

This review has several limitations that should be acknowledged. First, the exclusively qualitative design does not allow for quantification of the prevalence, severity, or relative weight of the identified phenomena, limiting its capacity for directly informing intervention thresholds (eg, optimal training duration) in nursing administration. Second, most included studies originated from countries with moderate-to-high digitalization and generally permissive AI policies, with underrepresentation of low-resource settings (eg, Southeast Asia, Africa, and Latin America) and regions with stringent AI regulation (eg, the European Union under the European Union AI Act). This predominantly policy-permissive sample may have overestimated acceptance, and generalizing to underrepresented settings requires caution. Third, as this review captures findings published up to June 2026, the timeliness of these findings varies: user typologies may become dated, ethical and professional concerns may intensify with deeper AI integration, and training needs will evolve from basic skills to advanced clinical applications. We have updated the search to include the latest literature and recommend regular updates of the review. Fourth, the restriction to English and Chinese publications may have excluded relevant studies in other languages, and despite search revisions, comprehensive coverage cannot be guaranteed. Finally, thematic saturation was not formally assessed, although data redundancy began to emerge after synthesizing the first ten studies [27-36]. Given the contextual heterogeneity of the included studies, additional research in underrepresented settings may yield new thematic dimensions. Regarding transferability, we have provided detailed contextual descriptors for each primary study (Multimedia Appendix 3) to facilitate readers’ assessment of applicability. However, the predominantly policy-permissive, digitized contexts may limit direct transferability to low-resource or highly regulated settings.

### Future Direction

This review provides an evidence base for developing context-aware guidelines that prioritize nurse agency and patient safety; future research could use longitudinal and mixed methods designs to track the coevolution of GenAI capabilities and nursing practice across diverse health care settings. Comparative studies across health care systems, cultural contexts, and policy environments are needed to better understand the contextual factors shaping GenAI adoption and its challenges. Given that the current evidence base is insufficient for systematic subgroup comparisons, future research should explicitly differentiate between various GenAI applications—ranging from administrative chatbots (eg, for drafting handover summaries) to clinical decision support tools and robotic systems. This would allow researchers to examine whether nurses’ perceptions, trust, and usage patterns differ systematically across these categories. Such granular insights are essential for tailoring implementation strategies, training programs, and governance frameworks to the specific risks and benefits of each tool type. Longitudinal studies are equally crucial for tracking how nurse experiences and attitudes transform alongside the technology itself.

### Conclusions

This systematic review synthesized qualitative evidence from 13 studies [24-36] involving 234 registered nurses from 6 countries. The findings reveal three interconnected dimensions shaping nurses’ engagement with GenAI: a spectrum of adoption patterns ranging from passive observation to pragmatic use and proactive integration; multidimensional perceived threats including functional deficits, professional erosion, and accountability ambiguity; and the moderating role of organizational readiness across cultural, technical, and institutional domains. These findings advance the TAM-AIN framework by demonstrating that organizational readiness moderates adoption by either alleviating perceived threats and fostering proactive adoption or reinforcing barriers and perpetuating conditional use. Accordingly, the unmet needs call for synchronized progress across workforce development, tool design, and institutional oversight, ensuring that efficiency gains do not compromise the humanistic essence of nursing care amid global workforce shortages.

## Supplementary material

10.2196/99489 Multimedia Appendix 1 Search strategy used in the literature search

10.2196/99489 Multimedia Appendix 2 Quality evaluation of the included literature

10.2196/99489 Multimedia Appendix 3 Data extraction table

10.2196/99489 Checklist 1 PRISMA checklist.

10.2196/99489 Checklist 2 ENTREQ checklist.

## Abbreviations

EHRs: electronic health records
ENTREQ: Enhancing Transparency in Reporting the Synthesis of Qualitative Research
GAN: generative adversarial network
GenAI: generative AI
GRADE-CERQual: Grading of Recommendations Assessment, Development and Evaluation-Confidence in the Evidence from Reviews of Qualitative research
JBI: Joanna Briggs Institute
LLMs: large language models
PICoS: Population, Phenomenon of Interest, Context, and Study Design
PRISMA: Preferred Reporting Items for Systematic Reviews and Meta-Analyses
PROSPERO: International Prospective Register of Systematic Reviews
TAM-AIN: Technology Acceptance Model for Artificial Intelligence in Nursing
VAEs: variational autoencoders
