# Supplementary material for: Nurses’ Attitudes and Experiences With Generative AI in Clinical Practice: Qualitative Systematic Review
Source: J Med Internet Res. 2026 Nov 6;28:e99489. doi: 10.2196/99489 (PMC13742299; doi:10.2196/99489)
Supplement: Supplementary file 1 [file jmir-v28-e99489-s001.docx]

| **1. Search strategy for PubMed** | |
| --- | --- |
| #1 | “Artificial Intelligence”[MeSH] OR “Generative Artificial Intelligence”[MeSH] OR “Generative AI”[Title/Abstract] OR “GenAI”[Title/Abstract] OR “GAI”[Title/Abstract] |
| #2 | “Artificial Intelligence Generated Content”[Title/Abstract] OR “AIGC”[Title/Abstract] |
| #3 | “Natural Language Processing”[MeSH] OR “large language model*”[Title/Abstract] OR “LLM”[Title/Abstract] OR “LLMs”[Title/Abstract] |
| #4 | “ChatGPT”[Title/Abstract] OR “ChatGPTs”[Title/Abstract] OR “Chat-GPT”[Title/Abstract] OR “Chat GPT”[Title/Abstract] |
| #5 | #1 OR #2 OR #3 OR#4 |
| #6 | “Nursing”[MeSH Terms] OR “nurses” OR “nurs*”[Title/Abstract] |
| #7 | “Health Personnel”[MeSH Terms] OR “health care personnel”[Title/Abstract] OR “medical personnel”[Title/Abstract] |
| #8 | #6 OR #7 |
| #9 | “Qualitative Research”[MeSH] OR “qualitative study”[Title/Abstract] OR “grounded theory”[Title/Abstract] OR “focus group”[Title/Abstract] OR “phenomenolog*”[Title/Abstract] OR “ethnograph*”[Title/Abstract] OR “interview”[Title/Abstract] OR “interviews”[Title/Abstract] OR “thematic analysis”[Title/Abstract] OR “content analysis”[Title/Abstract] OR “mixed method”[Title/Abstract] |
| #10 | #5 AND #8 AND #9 |
| Filters: from 2018/1/1 - 2026/6/30 761 | |
| **2. Search strategy for Web of Science Core Collection** | |
| #1 | TS=(“artificial intelligence” OR “generative artificial intelligence” OR “Generative AI” OR “GenAI” OR “GAI”) |
| #2 | TS=(“Artificial Intelligence Generated Content” OR “AIGC”) |
| #3 | TS=(“natural Language Processing” OR “large language model*” OR “LLM” OR “LLMs”) |
| #4 | TS=(“ChatGPT” OR “ChatGPTs” OR “Chat-GPT” OR “Chat GPT”) |
| #5 | #1 OR #2 OR #3 OR#4 |
| #6 | TS=(“nurs*” OR “Nurses” OR “Nursing”) |
| #7 | TS=(“Health Personnel” OR “health care personnel” OR “medical personnel”) |
| #8 | #6 OR #7 |
| #9 | TS=(“qualitative research” OR “qualitative study” OR “grounded theory” OR “focus group” OR “phenomenolog*” OR “ethnograph*” OR “interview” OR “interviews” OR “thematic analysis” OR “content analysis” OR “mixed method”) |
| #10 | #5 AND #8 AND #9 |
| Filters: 2018-01-01 to 2026-06-30 (Publication Date) Results: 518 | |
| **3. Search strategy for Cochrane Library** | |
| #1 | MeSH descriptor: [Artificial Intelligence] explode all trees |
| #2 | MeSH descriptor: [Generative Artificial Intelligence] explode all trees |
| #3 | MeSH descriptor: [Natural Language Processing] explode all trees |
| #4 | ((“Artificial Intelligence”) OR (“generative artificial intelligence”) OR (“Generative AI”) OR (“GenAI”) OR (“GAI”)):ti,ab,kw |
| #5 | ((“Artificial Intelligence Generated Content”) OR (“AIGC”)):ti,ab,kw |
| #6 | ((“natural Language Processing”) OR (large language model*) OR (“LLM”) OR (“LLMs”)):ti,ab,kw |
| #7 | ((“ChatGPT”) OR (“ChatGPTs”) OR ("Chat-GPT") OR (“Chat GPT”)):ti,ab,kw |
| #8 | #1 OR #2 OR #3 OR#4 OR #5 OR #6 OR#7 |
| #9 | MeSH descriptor: [Nursing] explode all trees |
| #10 | MeSH descriptor: [Health Personnel] explode all trees |
| #11 | ((nurs*) OR (“Nurses”) OR (“Nursing”)):ti,ab,kw |
| #12 | ((“Health Personnel”) OR (“health care personnel”) OR (“medical personnel”)):ti,ab,kw |
| #13 | #9 OR #10 OR #11 OR#12 |
| #14 | MeSH descriptor: [Qualitative Research] explode all trees |
| #15 | ((“qualitative research”) OR (“qualitative study”) OR (“grounded theory”) OR (“focus group”) OR (phenomenolog*) OR (ethnograph*) OR (“interview”) OR (“interviews”) OR (“thematic analysis”) OR (“content analysis” ) OR (“mixed method”)):ti,ab,kw |
| #16 | #14 OR #15 |
| #17 | #8 AND #13 AND #16 |
| Filters: 2018-01-01 to 2026-06-30 (first published) Results: 76 | |
| **4. Search Strategy for EBSCOhost CINAHL** | |
| #1 | AB=(“artificial intelligence” OR “generative artificial intelligence” OR “Generative AI” OR “GenAI” OR “GAI” OR “Artificial Intelligence Generated Content” OR “AIGC” OR “natural Language Processing” OR “large language model*” OR “LLM” OR “LLMs” OR “ChatGPT” OR “ChatGPTs” OR “Chat-GPT” OR “Chat GPT”) |
| #2 | AB=(“nurs*” OR “Nurses” OR “Nursing” OR “Health Personnel” OR “health care personnel” OR “medical personnel”) |
| #3 | AB=(“qualitative research” OR “qualitative study” OR “grounded theory” OR “focus group” OR “phenomenolog*” OR “ethnograph*” OR “interview” OR “interviews” OR “thematic analysis” OR “content analysis” OR “mixed method”) |
| #4 | #1 AND #2 AND #3 |
| Filters: 2018-01 to 2026-06 Results: 196 | |
| **5. Search Strategy for Embase (including MEDLINE)** | |
| #1 | 'Artificial Intelligence'/exp |
| #2 | 'Generative artificial intelligence'/exp |
| #3 | 'Natural Language Processing'/exp |
| #4 | 'artificial intelligence':ti,ab,kw OR 'generative artificial intelligence':ti,ab,kw OR 'generative ai':ti,ab,kw OR 'genai':ti,ab,kw OR 'gai':ti,ab,kw OR 'artificial intelligence generated content':ti,ab,kw OR 'aigc':ti,ab,kw OR 'natural language processing':ti,ab,kw OR 'large language model*':ti,ab,kw OR 'llm':ti,ab,kw OR 'llms':ti,ab,kw OR 'chatgpts':ti,ab,kw OR 'chat-gpt':ti,ab,kw OR 'chatgpt':ti,ab,kw |
| #5 | #1 OR #2 OR #3 OR #4 |
| #6 | 'Nursing'/exp |
| #7 | 'Health Personnel'/exp |
| #8 | 'nurs*':ti,ab,kw OR 'nurses':ti,ab,kw OR 'nursing':ti,ab,kw OR 'health personnel':ti,ab,kw OR 'health care personnel':ti,ab,kw OR 'medical personnel':ti,ab,kw |
| #9 | #6 OR #7 OR #8 |
| #10 | 'Qualitative Research'/exp |
| #11 | 'qualitative research':ti,ab,kw OR 'qualitative study':ti,ab,kw OR 'grounded theory':ti,ab,kw OR 'focus group':ti,ab,kw OR 'phenomenolog*':ti,ab,kw OR 'ethnograph*':ti,ab,kw OR 'interview':ti,ab,kw OR 'interviews':ti,ab,kw OR 'thematic analysis':ti,ab,kw OR 'content analysis':ti,ab,kw OR 'mixed method':ti,ab,kw |
| #12 | #10 OR #11 |
| #13 | #5 AND #9 AND #12 |
| Filters: 2018-01-01 to 2026-06-30 (Publication Date) Results: 2184 | |
| **6. Search Strategy for ProQuest** | |
| #1 | abstract(“artificial intelligence” OR “generative artificial intelligence” OR “Generative AI” OR “GenAI” OR “GAI” OR “Artificial Intelligence Generated Content” OR “AIGC” OR “natural Language Processing” OR “large language model*” OR “LLM” OR “LLMs” OR “ChatGPT” OR “ChatGPTs” OR “Chat-GPT” OR “Chat GPT”) |
| #2 | abstract(“nurs*” OR “Nurses” OR “Nursing” OR “Health Personnel” OR “health care personnel” OR “medical personnel”) |
| #3 | abstract(“qualitative research” OR “qualitative study” OR “grounded theory” OR “focus group” OR “phenomenolog*” OR “ethnograph*” OR “interview” OR “interviews” OR “thematic analysis” OR “content analysis” OR “mixed method”) |
| #4 | #1 AND #2 AND #3 |
| Additional limits - Date: From 2018 January 01 to 2026 June 30  Results: 233 | |
| **7. Search Strategy for Sinomed** | |
| #1 | "人工智能"[常用字段] OR "生成式人工智能"[常用字段] OR "大语言模型"[常用字段] OR "ChatGPT"[常用字段] OR "Chat-GPT"[常用字段] OR "Chat GPT"[常用字段] OR "Deepseek"[常用字段] OR "深度求索"[常用字段] |
| #2 | "护士"[常用字段] OR "护理"[常用字段] OR "医护"[常用字段] OR "医务人员"[常用字段] OR "医务工作者"[常用字段] |
| #3 | "质性研究"[常用字段] OR "定性研究"[常用字段] OR "焦点小组"[常用字段] OR "扎根理论"[常用字段] OR "现象学"[常用字段] OR "访谈"[常用字段] OR "主题分析"[常用字段] OR "内容分析"[常用字段] |
| #4 | #1 AND #2 AND #3 |
| Filters: 2018-01-01 to 2026-06-30  Results: 69 | |
| **8. Search Strategy for CNKI** | |
| #1 | 主题: 人工智能 + 生成式人工智能 + 大语言模型 + ChatGPT + Chat-GPT + Chat GPT + Deepseek + 深度求索 |
| #2 | 主题: 护士 + 护理 + 医护 + 医务人员 + 医务工作者 |
| #3 | 主题: 质性研究 + 定性研究 + 焦点小组 + 扎根理论 + 现象学 + 访谈 + 主题分析 + 内容分析 |
| #4 | #1 AND #2 AND #3 |
| Filters: 2018-01-01 to 2026-06-30 (first published) Results: 135 | |
| **9. Search Strategy for Wan Fang** | |
| #1 | 主题: "人工智能" OR "生成式人工智能" OR "大语言模型" OR "ChatGPT" OR "Chat-GPT" OR "Chat GPT" OR "Deepseek" OR "深度求索" |
| #2 | 主题: "护士" OR "护理" OR "医护" OR "医务人员" OR "医务工作者" |
| #3 | 主题: "质性研究" OR "定性研究" OR "焦点小组" OR "扎根理论" OR "现象学" OR "访谈" OR "主题分析" OR "内容分析" |
| #4 | #1 AND #2 AND #3 |
| Filters: 2018-01-01 to 2026-06-30 Results: 86 | |
| **10. Search Strategy for VIP** | |
| #1 | 摘要: "人工智能" OR "生成式人工智能" OR "大语言模型" OR "ChatGPT" OR "Chat-GPT" OR "Chat GPT" OR "Deepseek" OR "深度求索" |
| #2 | 摘要: "护士" OR "护理" OR "医护" OR "医务人员" OR "医务工作者" |
| #3 | 摘要: "质性研究" OR "定性研究" OR "焦点小组" OR "扎根理论" OR "现象学" OR "访谈" OR "主题分析" OR "内容分析" |
| #4 | #1 AND #2 AND #3 |
| Filters: 2018-01-01 to 2026-06-30 Results: 88 | |
