# Supplementary material for: Nurses’ Attitudes and Experiences With Generative AI in Clinical Practice: Qualitative Systematic Review
Source: J Med Internet Res. 2026 Nov 6;28:e99489. doi: 10.2196/99489 (PMC13742299; doi:10.2196/99489)
Supplement: Supplementary file 2 [file jmir-v28-e99489-s002.docx]

Quality evaluation of the included literature.

|  | **①** | **②** | **③** | **④** | **⑤** | **⑥** | **⑦** | **⑧** | **⑨** | **⑩** | Score (%) | Risk |
| --- | --- | --- | --- | --- | --- | --- | --- | --- | --- | --- | --- | --- |
| Nazir, M, et al. (2026)^[1]^ | Y | Y | Y | Y | Y | N | N | Y | Y | Y | 80% | Low |
| Shankar, R, et al.(2026)^[2]^ | Y | Y | Y | Y | Y | Y | Y | Y | Y | Y | 100% | Low |
| Qu, T. X, et al.(2026)^[3]^ | Y | Y | Y | Y | Y | N | N | Y | Y | Y | 80% | Low |
| Turki M Alanzi . (2023)^[4]^ | Y | Y | Y | Y | Y | N | N | Y | Y | Y | 80% | Low |
| Liu JB, et al. (2025)^[5]^ | Y | Y | Y | Y | Y | Y | Y | Y | Y | Y | 100% | Low |
| Artuwaili AN, et al. (2025)^[6]^ | Y | Y | Y | Y | Y | Y | Y | Y | Y | Y | 100% | Low |
| Kang RF, et al. (2025)^[7]^ | Y | Y | Y | Y | Y | Y | Y | Y | Y | Y | 100% | Low |
| Han J,et al.(2026)^[8]^ | Y | Y | Y | Y | Y | Y | Y | Y | Y | Y | 100% | Low |
| Bautista JR, et al. (2025)^[9]^ | Y | Y | Y | Y | Y | Y | Y | Y | Y | Y | 100% | Low |
| Tong L,et al. (2025)^[10]^ | Y | Y | Y | Y | Y | N | N | Y | Y | Y | 80% | Low |
| Ziteng Wang ,et al. (2025)^[11]^ | Y | Y | Y | Y | Y | N | Y | Y | Y | Y | 90% | Low |
| Binyan Yin ,et al. (2025)^[12]^ | Y | Y | Y | Y | Y | N | Y | Y | U | Y | 80% | Low |
| Temsah, M,et al. (2026)^[13]^ | Y | Y | Y | Y | Y | N | N | Y | Y | Y | 80% | Low |

Note:Y, yes; N, no; U, unclear.

①Is there congruence between the stated philosophical perspective and the research methodology? ② Is there congruity between the research methodology and the research question or objectives? ③ Is there congruity between the research methodology and the methods used to collect the data? ④ Is there congruity between the research methodology and representation and analysis of data? ⑤ Is there congruity between the research methodology and the interpretation of the results? ⑥ Is there a statement locating the researcher culturally or theoretically? ⑦ Is the influence of the researcher on the research, and vice versa, addressed? ⑧ Are the participants, and their voices , adequately represented? ⑨ Is the research ethical according to current criteria or, for recent studies, and is there evidence of ethical approval by an appropriate body? ⑩ Do the conclusions drawn in the research report flow from the analysis, or interpretation, of the data?

The criteria for determining the risk of bias were as follows: a score of ≥70% was classified as Low risk, 50%–69% as Moderate risk, and <50% as High risk.

**Reference**

[1] Nazir M，Carland J E，Keep M，et al. "...it saves so much time": A qualitative exploration of the use of Generative Artificial Intelligence by the health workforce[J]. Health Policy and Technology， 2026， 15(1).

[2] Shankar R，Lim A，Xu Q. Nurses' Lived Experiences of Generative Artificial Intelligence-Enabled Shift Handover Innovation: A Descriptive Phenomenological Study[J]. J Adv Nurs， 2026.

[3] 瞿田星，宋辉，王新月，等. 护理科研人员对生成式人工智能的认知与应用体验的质性研究[J]. 全科护理， 2026， 24(6): 1033-1038.

[4] Alanzi T M. Impact of ChatGPT on Teleconsultants in Healthcare: Perceptions of Healthcare Experts in Saudi Arabia[J]. Journal of multidisciplinary healthcare， 2023， 16: 2309-2321.

[5] Liu J，Wang L，He X，et al. Leveraging generative artificial intelligence to enhance ICU novice simulation instructors’ case design: A qualitative study[J]. Clinical Simulation in Nursing， 2025， 105: 101770.

[6] Alruwaili A N，Alshammari A M，Alhaiti A，et al. Neonatal nurses' experiences with generative AI in clinical decision-making: a qualitative exploration in high-risk nicus[J]. BMC nursing， 2025， 24(1): 386.

[7] Kang R，Xuan Z，Tong L，et al. Nurse Researchers' Experiences and Perceptions of Generative AI: Qualitative Semistructured Interview Study[J]. Journal of medical Internet research， 2025， 27: e65523.

[8] Han J，Ryu K H，Kim M，et al. Perceptions and Readiness for Generative Artificial Intelligence Implementation Among Oncology Nurses: A Qualitative Study in a Specialized Cancer Hospital[J]. Asian Nurs Res (Korean Soc Nurs Sci)， 2026， 20(1): 95-106.

[9] Bautista J R，Westendorf R，Goth O. Using a Socio-Technical Strategy to Identify the Use and Implications of Generative Artificial Intelligence Tools on Nursing Education and Practice[J]. Journal of advanced nursing， 2025: 10-1111.

[10] Tong L，Niu Y，Zhou L，et al. Perceptions and experiences of generative artificial intelligence training to support research for Chinese nurses: A qualitative focus group study[J]. International journal of nursing sciences， 2025， 12(3): 210-217.

[11] 王孜腾，佟玲，康瑞赋，等. 母婴护理人员对生成式人工智能的使用体验[J]. 中国护理管理， 2025， 25(3): 421-425.

[12] 殷彬燕，彭焕椽，朱丽瑜，等. 医护人员对类ChatGPT语言模型在医学领域中应用的认知和实践的质性研究[J]. 全科护理， 2025， 23(7): 1378-1381.

[13] Temsah M，Abouammoh N，Alsatrawi M，et al. Exploring Early Perceptions and Experiences of ChatGPT in Pediatric Critical Care: A Qualitative Study Among Health Care Professionals[J]. Clinical pediatrics， 2026， 65(1): 11-21.
