# Supplementary material for: Nurses’ Attitudes and Experiences With Generative AI in Clinical Practice: Qualitative Systematic Review
Source: J Med Internet Res. 2026 Nov 6;28:e99489. doi: 10.2196/99489 (PMC13742299; doi:10.2196/99489)
Supplement: Supplementary file 3 [file jmir-v28-e99489-s003.docx]

Data extraction table：study characteristics

| **Author (year)** | **Country** | **Setting** | **Study design** | **Data collection Data analysis** | **Participants** | **Type of** Generative AI **and their key features** | **Phenomenon of interest** | **Main findings** |
| --- | --- | --- | --- | --- | --- | --- | --- | --- |
| Nazir, M, et al. (2026)^[1]^ | Australia | multiple healthcare organizations and facilities in Australia | mixed-methods study | semi  structured interviews;free-text thematic analysis | Ten health professionals participated in interviews  (3 nurses) | ChatGPT , this was followed by Bard and Copilot. Ada Doctor, Glass AI, Mac Whisperer, Claude, and an institution-specific tool were identified by indi  vidual participants. | to understand how health professionals are using emerging Gen AI tools, such as chatbots, in their work, and their perceptions of the role of Gen AI in healthcare in the coming years. | Four themes were identified:  ①Gen AI tools help health professionals reduce their workload  ②Barriers to Gen AI use align with perceived risks, trust and the lack of organizational support  ③Technological, individual and organisation factors drive health professionals’ use of  Gen AI  ④Gen AI tools won’t replace humans, but could augment their capabilities |
| Shankar, R, et al.(2026)^[2]^ | Singapore | Alexandra Hospital ; participants had ≥4 weeks of direct handover experience with the AI system (CareScribe). | Descriptive phenomenological study | Semi‑structured individual interviews and focus group discussions ; Colaizzi’s seven‑step method | 18 registered nurses | CareScribe – a generative AI tool that uses voice recognition and GPT‑4 to capture, summarise, and structure nursing handover information | Nurses’ lived experiences of a generative AI‑enabled shift handover innovation | Five themes were identified:   1. burden of fragmented documentation 2. navigating technological change with cautious optimism 3. anchoring innovation in familiar clinical frameworks; 4. anticipating barriers to seamless integration 5. envisioning enhanced patient safety and professional practice. |
| Qu, T. X, et al.(2026)^[3]^ | China | Three tertiary grade A hospitals | Descriptive qualitative study | Semi‑structured in‑depth individual interviews; Colaizzi’s 7‑step method | 14 nursing researchers (all female, aged 26–47 years, experience 2–25 years) (14 nurse) | Generative AI tools (e.g., ChatGPT, Wenxin Yiyan, Gemini) | Nursing researchers’ real perceptions, practical experiences, and ethical considerations regarding the application of generative AI in nursing research. | Four themes were identified:   1. Enhancing research efficiency and capability 2. Risks and ethical dilemmas 3. Competence and role reconstruction 4. Future pathways for norms and professional development |
| Turki M Alanzi . (2023)^[4]^ | Saudi Arabia | Hospitals | Descriptive qualitative study | Qualitative Study; focus group discussions;  Thematic analysis | 54 healthcare professionals (24 nurses) | ChatGPT；large language model, natural language processing, conversational AI, multilingual support | To investigate the impact of ChatGPT on teleconsultants in managing their operations and services | Positive impact: ① Informational support; ② Diagnostic assistance; ③ Communication; ④ Enhancing efficiency; ⑤ Cost and time saving; ⑥ Personalizing care; ⑦ Multilingual support; ⑧ Assisting in medical research; ⑨ Decision-making; ⑩ Documentation; ⑪ Continuing education; ⑫ Enhancing team collaboration.  Challenges: ① Misdiagnosis and errors; ② Issues in personalized care; ③ Limited medical context/knowledge; ④ Communication challenges; ⑤ Increased dependency on technology; ⑥ Ethical and legal issues. |
| Liu JB, et al. (2025)^[5]^ | China | Hospitals | Descriptive qualitative study | Qualitative Study; semi-structured interviews; Thematic analysis | 13 ICU novice simulation instructors (13 nurses) | Generative Artificial Intelligence (Gen AI)/ChatGPT: large language model, generates structured simulation cases based on prompts | To explore ICU novice simulation instructors' experiences of using Gen AI for simulation case design | Three themes were identified:  ① Perceived value and benefits;  ② Potential for expansion;  ③ Concerns and limitations. |
| Artuwaili AN, et al. (2025)^[6]^ | Saudi Arabia | Hospitals (NICUs) | Descriptive qualitative study | Qualitative Study; semi-structured interviews and focus groups; Thematic analysis | 33 neonatal nurses (33 nurses) | Generative AI: analyzes complex datasets, predicts neonatal sepsis, suggests ventilation adjustments, recommends feeding protocols, provides real-time risk identification | To explore neonatal nurses' experiences with generative AI in clinical decision-making in high-risk NICUs | Five themes were identified:  ① Clinical Decision-Making;  ② Professional Practice Transformation;  ③ Organizational Factors;  ④ Cultural Influences;  ⑤ Implementation Challenges. |
| Kang RF, et al. (2025)^[7]^ | China | Hospitals; Academic institutions | Descriptive qualitative study | Qualitative Study; semi-structured interviews;  Inductive content analysis | 27 nurse researchers (27 nurses) | Generative AI (e.g., ChatGPT, ERNIE Bot): large language models, text-to-text and text-to-image generation, capable of assisting in research tasks | To describe the experiences and perceptions of generative AI among Chinese nurse researchers | Five themes were identified:  ① Diverse reflections on human-machine symbiosis;  ② Multiple factors of the usage experience;  ③ Research paradigm reshaping in the infancy stage;  ④ Application risks of generative AI;  ⑤ The co-improvement of technology and literacy. |
| Han J,et al.(2026)^[8]^ | South Korea | Hospitals (Specialized Cancer Hospital) | Descriptive qualitative study | Qualitative Study; in-depth interviews;Colaizzi's phenomenological analysis | 17 oncology nurses  (17 nurses) | Generative AI (Gen AI), specifically including large language models/chatbots (e.g., OpenAI's GPT) applied to nursing tasks | To explore oncology nurses' perceptions and readiness for generative AI implementation in a specialized cancer hospital | Three themes were identified:  ① Change management factors;  ② Limitations of change management;  ③ Preparation strategies for change management. |
| Bautista JR, et al. (2025)^[9]^ | USA | Hospitals; Academic institutions | Descriptive qualitative study | Qualitative Study; interviews;  Framework Method | 32 nursing stakeholders (32 nurses) | Generative AI tools (ChatGPT, Copilot, Perplexity, Gemini, etc.):  text generation, information seeking,  content creation, virtual assistance | To identify the use and implications of generative AI tools on nursing education and practice using a socio-technical strategy | Four themes were identified:  ① Use cases of GenAI tools for nursing education and practice;  ② Implications of using GenAI tools on nursing education;  ③ Implications of using GenAI tools on nursing practice;  ④ GenAI capacity-building efforts. |
| Tong L,et al. (2025)^[10]^ | China | Training institution | Descriptive qualitative study | Focus group interviews;  Conventional content analysis | 23 clinical nurses  (23 nurses) | Generative AI (Nurse AI, Kimi Chat):  text generation, research assistance (topic selection, literature review, proposal writing) | To explore clinical nurses' perceptions and experiences of GAI training to support research | Four themes were identified:  ① Utilization efficacy: cope with research ability, affected by many factors;  ② Booster research: growth and challenges go hand in hand;  ③ Role reversal: from GAI-dominated to nurse-dominated;  ④ Beautiful dream: more features on research, more assistants on clinical care. |
| Ziteng Wang et al. (2025)^[11]^ | China | Hospitals | Qualitative study | Semi-structured interviews; Content analysis | 16 maternal and infant care professionals (16 nurses) | Generative AI:  text generation, information provision,  creative work assistance | To explore the experience of maternal and infant care professionals in using Generative AI | Three themes were identified:  ① The sense of positive effects of use;  ② Constraints of use;  ③ Needs and expectations for use. |
| Binyan Yin et al. (2025)^[12]^ | China | Hospitals | Qualitative study | Semi-structured interviews; Colaizzi's analysis | 21 healthcare professionals (12 nurses) | ChatGPT-like language models: trained on massive data, capable of Q&A, writing, translation, and assisting in clinical, research, and educational tasks | To explore the cognition and practice of healthcare professionals regarding the application of ChatGPT-like language models in the medical field | Three themes were identified:  ① Different levels of understanding;  ② Different attitudes of acceptance;  ③ Unsatisfactory actual usage. |
| Temsah, M. et al. (2026)^[13]^ | Saudi Arabia | Hospital；two PICUs | Descriptive qualitative study | Qualitative Study;  focus group discussions;  Thematic analysis | 18 healthcare professionals (2 nurses) | ChatGPT: large language model, conversational AI, natural language processing, multi-language support | explores HCPs' initial impressions and experiences using ChatGPT, a Generative Pre-trained Transformer, in Pediatric Critical Care Units (PICUs) | Three themes were identified:  ① Familiarity and usability of ChatGPT;  ② Its role in clinical and organizational tasks;  ③ Ethical concerns |

**参考文献**

[1] Nazir M，Carland J E，Keep M，et al. "...it saves so much time": A qualitative exploration of the use of Generative Artificial Intelligence by the health workforce[J]. Health Policy and Technology， 2026， 15(1).

[2] Shankar R，Lim A，Xu Q. Nurses' Lived Experiences of Generative Artificial Intelligence-Enabled Shift Handover Innovation: A Descriptive Phenomenological Study[J]. J Adv Nurs， 2026.

[3] 瞿田星，宋辉，王新月，等. 护理科研人员对生成式人工智能的认知与应用体验的质性研究[J]. 全科护理， 2026， 24(6): 1033-1038.

[4] Alanzi T M. Impact of ChatGPT on Teleconsultants in Healthcare: Perceptions of Healthcare Experts in Saudi Arabia[J]. Journal of multidisciplinary healthcare， 2023， 16: 2309-2321.

[5] Liu J，Wang L，He X，et al. Leveraging generative artificial intelligence to enhance ICU novice simulation instructors’ case design: A qualitative study[J]. Clinical Simulation in Nursing， 2025， 105: 101770.

[6] Alruwaili A N，Alshammari A M，Alhaiti A，et al. Neonatal nurses' experiences with generative AI in clinical decision-making: a qualitative exploration in high-risk nicus[J]. BMC nursing， 2025， 24(1): 386.

[7] Kang R，Xuan Z，Tong L，et al. Nurse Researchers' Experiences and Perceptions of Generative AI: Qualitative Semistructured Interview Study[J]. Journal of medical Internet research， 2025， 27: e65523.

[8] Han J，Ryu K H，Kim M，et al. Perceptions and Readiness for Generative Artificial Intelligence Implementation Among Oncology Nurses: A Qualitative Study in a Specialized Cancer Hospital[J]. Asian Nurs Res (Korean Soc Nurs Sci)， 2026， 20(1): 95-106.

[9] Bautista J R，Westendorf R，Goth O. Using a Socio-Technical Strategy to Identify the Use and Implications of Generative Artificial Intelligence Tools on Nursing Education and Practice[J]. Journal of advanced nursing， 2025: 10-1111.

[10] Tong L，Niu Y，Zhou L，et al. Perceptions and experiences of generative artificial intelligence training to support research for Chinese nurses: A qualitative focus group study[J]. International journal of nursing sciences， 2025， 12(3): 210-217.

[11] 王孜腾，佟玲，康瑞赋，等. 母婴护理人员对生成式人工智能的使用体验[J]. 中国护理管理， 2025， 25(3): 421-425.

[12] 殷彬燕，彭焕椽，朱丽瑜，等. 医护人员对类ChatGPT语言模型在医学领域中应用的认知和实践的质性研究[J]. 全科护理， 2025， 23(7): 1378-1381.

[13] Temsah M，Abouammoh N，Alsatrawi M，et al. Exploring Early Perceptions and Experiences of ChatGPT in Pediatric Critical Care: A Qualitative Study Among Health Care Professionals[J]. Clinical pediatrics， 2026， 65(1): 11-21.
